# Experiences of participant and public involvement in an international randomized controlled trial for people living with dementia and their informal caregivers

**DOI:** 10.1186/s40900-024-00574-2

**Published:** 2024-05-02

**Authors:** Jodie Bloska, Sarah Crabtree, Nina Wollersberger, Oti Mitchell, Jenny Coles, Caroline Halsey, Geraldine Parry, Robert Stewart, Susan Thacker, Mark Thacker, Leica Claydon-Mueller, Yvette Winnard, Kate McMahon, Carina Petrowitz, Agnieszka Smrokowska-Reichmann, Beatrix van Doorn, Felicity A. Baker, Laura Blauth, Anna A. Bukowska, Karette Stensæth, Jeanette Tamplin, Thomas Wosch, Helen Odell-Miller

**Affiliations:** 1 Cambridge Institute for Music Therapy Research, Anglia Ruskin University, Cambridge, UK; 2 Public Contributor, Cambridge Institute for Music Therapy Research, Anglia Ruskin University, Cambridge, UK; 3 School of Allied Health and Social Care, Faculty of Health, Medicine and Social Care, Anglia Ruskin University, Cambridge, UK; 4 Faculty of Fine Arts and Music, The University of Melbourne, Melbourne, Australia; 5 Institute for Applied Social Sciences, Technical University of Applied Sciences Würzburg-Schweinfurt, Würzburg, Germany; 6 Institute of Applied Sciences, University of Physical Education in Kraków, Kraków, Poland; 7 Singing in Elderly Care, Singing Norway, Oslo, Norway; 8 Public Contributor, Centre for Research in Music and Health, Norwegian Academy of Music, Oslo, Norway; 9 Centre for Research in Music and Health, Norwegian Academy of Music, Oslo, Norway

**Keywords:** Dementia, PPI, Patient and public involvement, Caregivers, Music therapy, Music interventions, Occupational therapy, Psychosocial interventions, Randomized controlled trial

## Abstract

**Background:**

This study was initiated and co-designed by a Participant and Public Involvement (PPI) group attached to HOMESIDE, a randomized controlled trial that investigated music and reading interventions for people living with dementia and their family caregivers across five countries: Australia, Germany, Norway, Poland, and the UK. The aim was to capture experiences of PPI across the five countries, explore the benefits and challenges of PPI in dementia research, and identify contributions made to the study.

**Methods:**

We surveyed PPI members and academic researchers who collaborated on the HOMESIDE study. The survey was co-designed through consultation with PPI members and academics, alongside a small scoping literature review. Survey questions covered four topics: (1) expectations for PPI, (2) perceived contributions of PPI to the research study, (3) benefits and challenges of PPI, and (4) recommendations for future PPI in dementia research.

**Results:**

There were 23 responses, representing 50% of the PPI members (*n* = 16) and 29% of academics (*n* = 7). PPI was found to be beneficial to the research and individuals involved. Contributions to the research included supporting recruitment and publicity, advising on the design of participant-facing materials, guiding the design and delivery of the interventions, and identifying cultural differences affecting research delivery. PPI members benefited from building connections, sharing experiences and receiving support, learning about dementia and research, and gaining new unexpected experiences. Academics learned about the realities of living with dementia, which they felt informed and grounded their work. Several challenges were identified, including the need for clear expectations and objectives, inconsistency of PPI members across research stages, limitations of meeting online versus in-person, scheduling difficulties, and language barriers.

**Conclusions:**

This study identifies important considerations for implementing PPI within dementia studies and international healthcare research more broadly. Our findings guided the development of five recommendations: (1) involve PPI members as early as possible and throughout the research process; (2) create a space for constructive criticism and feedback; (3) have clear tasks, roles, and expectations for PPI members; (4) involve PPI members with a diverse range of experiences and backgrounds; and (5) embed infrastructure and planning to support PPI.

**Supplementary Information:**

The online version contains supplementary material available at 10.1186/s40900-024-00574-2.

## Plain language summary

Participant and Public Involvement (PPI) brings the knowledge of those with lived experience into research to improve research relevance and delivery. Our international study, called HOMESIDE, explored the benefits of music and reading activities for people with dementia and their family caregivers across five countries: Australia, Germany, Norway, Poland, and the UK. The study’s PPI members included people with dementia, family carers, and healthcare professionals, who met regularly with the research team throughout the 3-year study. The current article reports the findings of a co-designed survey about PPI within HOMESIDE. Initiated by the HOMESIDE PPI members, we carried out a survey of PPI members and academics who worked on the study to learn about the unique experiences, perspectives, and contributions of PPI across the international research team.

Our findings show that PPI helped to publicize the study, improved recruitment of research participants, and informed delivery of the interventions. Another important outcome was learning from each other; PPI members learned about dementia research and academics learned about the realities of living with dementia. However, the survey also highlighted challenges, including managing expectations, scheduling difficulties, and language barriers.

To support PPI in future dementia research, we highlight five recommendations: (1) involve PPI members as early as possible and throughout the research process; (2) create a space for constructive criticism and feedback; (3) have clear tasks, roles, and expectations for PPI members; (4) involve PPI members with a diverse range of experiences and backgrounds; and (5) embed infrastructure and planning to support PPI.

**Supplementary Information:**

The online version contains supplementary material available at 10.1186/s40900-024-00574-2.

## Background

Public involvement is essential to health and social care research, recognizing the importance of involving those with lived experience in guiding research. The term ‘Patient and Public Involvement’ is the standard term used within health and social care; however, the current study uses *Participant* and Public Involvement (PPI) to better represent the population that was involved, which included people with dementia and their family caregivers residing at home. PPI is defined as an active partnership with the public to influence and guide research, differentiating it from engagement in research dissemination (i.e., learning about research findings) or research participation (i.e., taking part in a research study) [1]. The ambition of PPI is to ensure research is meaningful and easily translated into practice, as well as to improve research quality and priorities by highlighting the perspectives of those with lived experience [2]. PPI also supports clinical research to be people-centered [3], which makes it easier for potential participants to take part and improves the experience of research participation.

Challenges in implementing PPI need to be identified and addressed to support public involvement that is not tokenistic [4, 5] and does not inadvertently contribute to increased health inequalities [6] or compromise the robustness of research [7, 8]. It is important to acknowledge that approaches to PPI can vary across countries and research teams due to organizational cultures [9, 10], access to funding [11], assumptions regarding the expertise or abilities of those living with a condition [10, 12], and how established PPI is within a country [13, 14]. PPI can also be applied in different ways depending on the stage of research and to allow flexibility in engaging members of the public [4, 8, 15, 16]. When identifying areas for PPI input, academics also need to consider the training needs of PPI members [17].

### Previous literature reporting PPI outcomes

There is a need to evaluate PPI, and to develop a consensus about best practices to ensure that PPI members’ and academics’ efforts, time, and resources are not fruitless [18, 19]. The research stage where PPI is most frequently reported is within the identification of research questions or priorities [14], rather than during the stages of research design, delivery, or analysis. Previously reported impacts of PPI in research studies are broad, ranging from impact on funding decisions to dissemination and policy change [4, 7]. Therefore, outcomes of PPI can be wide-ranging and unpredictable, depending on the stage of research, the approach taken, and the needs of the research context. It has also been highlighted that measuring impact against specific outcomes may not capture the important learning process that might occur between those with lived experience and academic researchers [16]. There may also be psychological benefits for PPI members by enabling those affected by a condition to contribute to change in services and policies [15]. Russell et al. recommend continuous reflexive evaluation to explore not only the impact of PPI on research but also the complexities, potential negative consequences, and power dynamics at play within public involvement in research [6, 20].

The current study aimed to evaluate the implementation and outcomes of PPI as part of an international study carried out in Australia, Germany, Norway, Poland, and the UK. The aim was to capture the contributions of PPI to the research study and to explore PPI members’ and academics’ experiences to identify any benefits and challenges that arose. This research provides an example of PPI, which can support better PPI implementation in future dementia research and health research more broadly, valuing PPI members’ experiences and feedback to enable more accessible public involvement in future research.

#### Study context

The current study evaluates PPI within the HOMESIDE (home-based family caregiver-delivered music and reading interventions for people living with dementia) randomized controlled trial (RCT). HOMESIDE investigated the effectiveness of a 12-week music intervention for people living with dementia and their informal caregivers compared to a reading intervention (active control) and usual care (control)^1^ [21]. The music and reading interventions were delivered by caregivers who were trained and supported by qualified healthcare professionals such as music therapists and occupational therapists. The 3-year RCT began in 2019, prior to the COVID-19 pandemic; restrictions implemented for public health from March 2020 impacted the trial, leading to a change in the protocol to carry out the study online rather than in-person. This also impacted PPI within the study, where meetings moved from in-person to online throughout most of the trial delivery. At the time of the current PPI study, the HOMESIDE trial was in its final stages of intervention delivery and data collection.

### PPI in HOMESIDE

PPI was embedded in the HOMESIDE trial from its inception and throughout the study, supporting design, delivery, and dissemination. In the development stages of the funding proposal, consultations took place with people with dementia, caregivers, and dementia day care staff to inform the development of the interventions and the research design. Upon successfully acquiring funding, formalized PPI advisory groups were established in each of the five countries to advise on the trial as it was rolled out and evaluated. PPI membership was reasonably consistent throughout; however, in some countries, some members resigned and some new members joined during the trial. Members were recruited through partnerships with the research team, previous research participants, and national organizations (such as the Norwegian Health Association, Singing Norway, Alzheimer Gesellschaft [Germany], Alzheimer's Association [Poland], Alzheimer Poland, Step Up for Dementia Research [Australia], and Alzheimer’s Society [UK]) who helped recruit volunteers via their networks. The groups included people living with dementia, family caregivers, and healthcare professionals working in the field of dementia care (see Table 1). PPI members were recruited as volunteers, with no financial compensation, although travel expenses for meetings were reimbursed.

**Table 1:** The HOMESIDE trial’s PPI membership makeup across national groups

Country	People living with dementia	Family caregivers	Professionals	Total
Australia	1	5	2	8
Germany	1	2	3	6
Norway	1	2	2	5
Poland	0	0	5	5
UK	1	6	1	8
**Total**	**4 (12.5%)**	**15 (46.9%)**	**13 (40.6%)**	**32 (100%)**

National PPI groups in each country met quarterly with their local study teams, focusing on study delivery within their respective countries. Meetings were often formal with an agenda, although research teams also held informal PPI gatherings to build rapport (e.g., lunch meetings). These meetings were a mixture of in-person, online, and hybrid. As part of their involvement, PPI members were also invited to attend international team meetings and research presentations. An international PPI group, comprised of country representatives from each national group, was led by the UK and chaired by a UK PPI member. The international group met twice yearly online to feedback from national groups, share ideas cross-culturally, and identify cultural differences and similarities.

As outlined in the original research funding application, the aims for the PPI groups were to:

- Highlight procedural issues for the study from the perspective of those with lived experience.
- Input into all aspects of the trial, including wording of recruitment invitations, plain language statements, consent forms, qualitative interview questions, and any participant-facing materials.
- Be involved in research tasks where appropriate.
- Gain insights into the interpretation of the study findings from those with lived experience.
- Determine suitable opportunities for disseminating the research results.

Many of the topics covered in national and international PPI meetings met these aims. Topics raised in national PPI groups were often brought to the international group for further discussion. These included:

- Advising on the transition to online delivery during the COVID-19 pandemic, such as sharing strategies for engaging participants over the screen and addressing potential challenges. Academic researchers had concerns about this transition, but PPI members in most countries encouraged the research to go online based on their own experiences.
- Reviewing and amending participant-facing materials such as the HOMESIDE website [22] and recruitment materials (for an example, see Additional file 1).
- Sharing new ideas for recruiting participants, and problem-solving issues arising in recruitment, eligibility, and retention. Some strategies discussed were implemented across all countries, such as the co-production of a recruitment video in each country’s language (see Additional file 2).
- Sharing own experiences of using music or reading, including the HOMESIDE interventions.
- Critical consideration of the best ways to encourage contributions from PPI members during meetings, including preferences for clear tasks, direct questions from academics, and strategies for including non-English speakers in international meetings. Discussions also centered on a need to expand PPI membership to be more diverse and include more people with a diagnosis of dementia. Strategies to try to reach these groups in different countries were discussed.
- Discussing long-term aspirations for future music therapy research in the field of dementia post-study completion. Some PPI members were involved in early discussions for related projects, such as the development of a mobile application based on the HOMESIDE music intervention [23]. These discussions also included considerations of the wider implications of HOMESIDE findings.
- Considering ways to share experiences of participating in the HOMESIDE PPI and encouraging others to get involved in future PPI opportunities. Early discussions revolved around what the best format, methods, and purpose of this output would be, ultimately resulting in this paper.

## Methods

The purpose of the current research was to explore the experiences of PPI during the HOMESIDE trial from both the PPI members’ and the academics’ perspectives. A cross-sectional survey was carried out to answer the following research questions:

1. What are PPI members’ and academic researchers’ experiences of PPI within a large-scale clinical trial of psychosocial interventions for dementia?
  - What are the benefits of implementing PPI?
  - What are the challenges of implementing PPI?
2. What are the HOMESIDE PPI members’ and academics’ recommendations for embedding PPI in future dementia research?

Therefore, the survey questions fell under the following themes: (1) expectations for PPI, (2) perceived contributions of PPI to the research study, (3) benefits and challenges of PPI, and (4) recommendations for future PPI in dementia research. The survey methods and results are reported in this paper following the survey reporting checklist developed by Kelley et al. [24] (see Additional file 3) and the Guidance for Reporting Involvement of Patients and the Public (GRIPP2) checklist [25] (see Additional file 4).

### PPI in the current study

The current study was initiated by HOMESIDE PPI members in the UK after discussing their own experiences of being involved in the trial and recognizing that their experiences may have differed from PPI members in the other countries. The UK PPI members suggested an anonymous international survey to encourage honest and open feedback about PPI experiences, expectations, and contributions during the trial. The survey questions were developed by PPI members and refined in conversation with academics. One of the UK PPI members (OM) responded to a call for a PPI member to work on the survey; she was paid an hourly rate as a co-researcher and was involved in the design and dissemination of the survey.

### Participants

Survey participants included HOMESIDE PPI members across all five countries (including people with dementia, family caregivers of people with dementia, and healthcare professionals) and academic researchers employed within the international HOMESIDE research team (including research assistants, PhD students, research fellows, postdoctoral researchers, and professors). We surveyed PPI members and academics who worked on HOMESIDE to gain feedback on the specific working practices and outcomes of the trial’s PPI implementation.

### Survey design and development

An online questionnaire-based survey [26] was developed and included two sets of survey questions: one for PPI members (see Additional file 5) and one for academics (see Additional file 6). The survey questions were developed through an iterative process, with initial questions suggested by the UK PPI members and then further refined based on previous literature and in consultation with representatives from the international research team, including both academics and PPI members. This process included sending the draft questions via e-mail for feedback and then discussion within a group meeting, as well as piloting the online survey to ensure it was user-friendly. The survey questions were split into four sections:

1. Background (PPI members only): Questions about participants’ connections to dementia, how they came to be involved in the study, and why they decided to take part in the PPI group.
2. Experience and contributions: Questions about how involved PPI groups were in the study, what their expectations were, and what they felt PPI contributed to the study.
3. Effects of PPI: Questions about how PPI impacted participants personally and professionally.
4. Future of PPI: Questions about the barriers and facilitators for the HOMESIDE PPI groups and recommendations for future PPI.

The survey questions were developed in English. The questions for PPI members were then translated into Norwegian, Polish, and German by a fluent speaker and circulated in the appropriate national language to members from each country. The questions for academics were circulated in English only.

### Data collection

The survey questions were entered into the ‘Online Surveys’ platform [27] for circulation. A representative from each country disseminated the survey invitation to their national PPI members. The first author disseminated the invitation to the academics working on the study. Potential participants received invitations to participate in the survey via e-mail in June 2022 and September 2022, with reminders to complete the survey in meetings between these dates. The survey was anonymous and due to the small number of potential participants and the risk of participant identification, demographic data were not collected. There was no remuneration for participation, and there was no obligation to take part. Before completing the survey, participants were given a participant information sheet and had to confirm their consent to take part. Ethical approval was obtained from the Anglia Ruskin University Cambridge School of Creative Industries School Research Ethics Panel in May 2022.

### Survey analysis

When the survey had closed, the survey data were downloaded from the Online Surveys platform and uploaded into Microsoft Excel for analysis. Responses from the Norwegian, Polish, and German participants were translated into English prior to analysis. The translations were undertaken by fluent speakers who were independent from the participants’ country’s research team to maintain anonymity and remove potential bias.

Quantitative data were analyzed using descriptive statistics including frequency distribution. Free-text responses were uploaded into NVivo qualitative data analysis software and analyzed thematically using a general inductive approach [28], which included the following stages: (1) initial reading of the text, (2) identification of specific segments related to objectives, (3) labeling the segments to create categories, (4) reduction of the categories to reduce overlap and redundancy, and (5) creating themes incorporating the most important categories. To check for coding consistency, four researchers coded the free-text responses separately before meeting to refine categories and develop the themes as a group. The identified themes are presented using illustrative quotes from participants.

## Results

The survey received a total of 23 responses: 16 from PPI members and 7 from academics on the HOMESIDE trial. This represents 50% of the PPI members on the HOMESIDE study and 29% of the academics working on the study. Survey responses from the PPI members were received in English (*n* = 9), German (*n* = 2), Norwegian (*n* = 2), and Polish (*n* = 3). All academic responses were received in English. PPI respondents consisted of individuals with a personal connection to dementia (i.e. living with dementia or family caregiver) (*n* = 8), healthcare professionals (*n* = 5), and those with both personal and professional connections to dementia (*n* = 3).

### Expectations

**Fig. 1:**
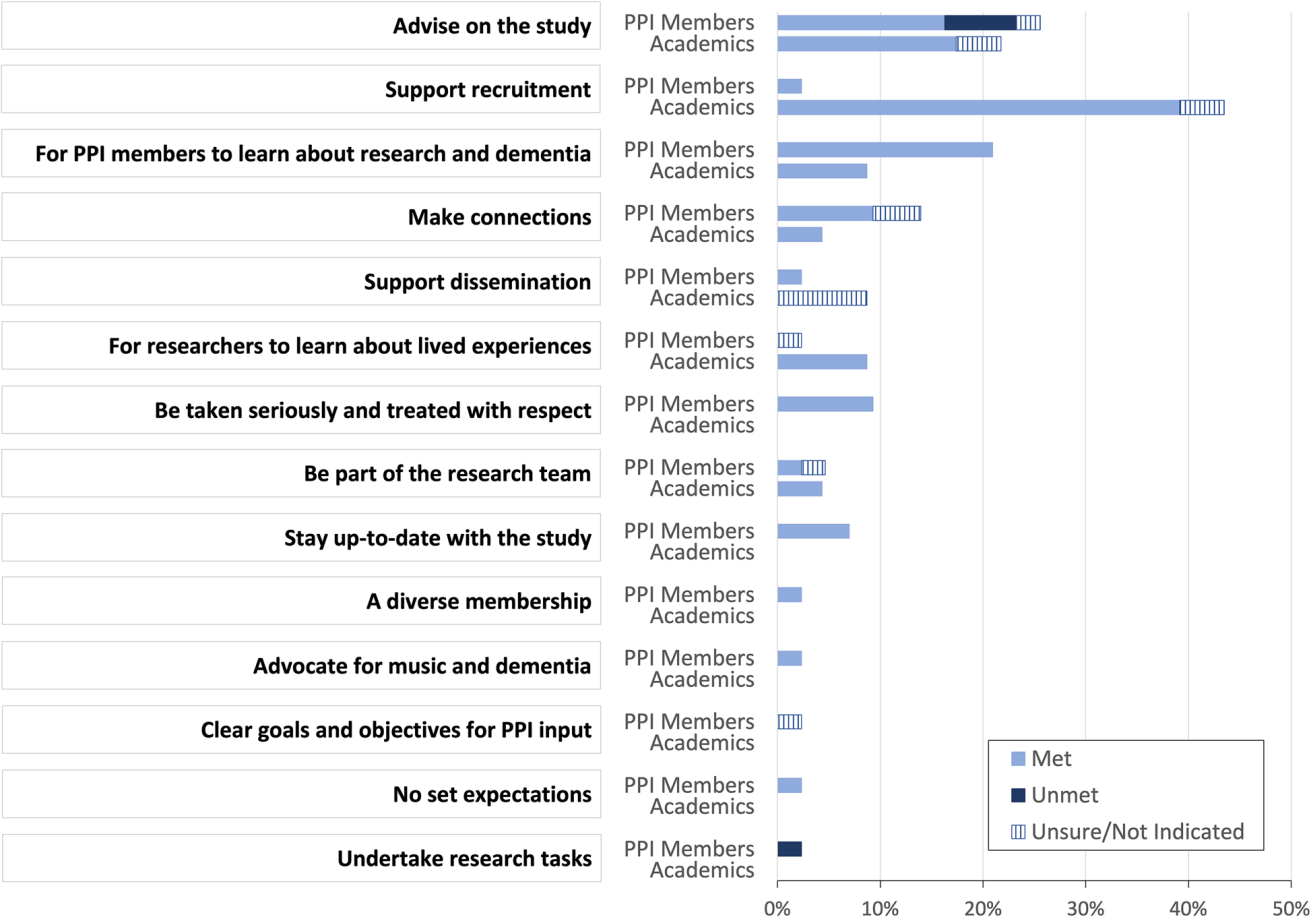
PPI members’ and academics’ self-reported expectations for PPI in HOMESIDE. Respondents indicated their expectations for PPI in the HOMESIDE trial and whether they were met or not. Sixty-six expectations were reported (43 by PPI members and 23 by academics), which were pooled into the 14 categories presented here. Percentages reported are based on the total reported expectations within each group, indicating where they were met or not met

Respondents’ self-reported expectations of PPI within the HOMESIDE trial are presented in Fig. 1. Respondents were asked to write their own expectations in a free-text field and then to indicate whether these were met or unmet during their involvement. A total of 66 expectations were reported (43 reported by PPI members and 23 reported by academics), which were then pooled into 14 categories.

The most indicated expectation for PPI members was ‘Advise on the study’, with a quarter of PPI members’ expectations (25.6%) relating to this topic. This category included responses that indicated broader expectations for sharing opinions and maintaining good ethical standards but also more specific contributions to the design of the study and materials. Academics most often indicated ‘supporting recruitment’ as an expectation, with nearly half of academics’ expectations (43.5%) relating to recruitment, including signposting to organizations, reaching new groups of potential participants, and feeding back on recruitment materials and methods. Supporting recruitment was far less indicated by PPI members, with only one PPI member reporting this.

Most of the expectations were met, with academics indicating 82.6% as met and PPI members reporting 76.7% as met. However, the expectation participants most often indicated as ‘Not Met’ or ‘Unsure’ was ‘advise on the study’. Participants indicated that this was due to the timeline of participants joining the PPI advisory group, after the funding was secured for the project, which left little room for their ability to change the design of the study or intervention specifically.

### Contributions to the study

Responses to questions related to the perceived level of contribution of PPI to the study are presented in Table 2. Academics surveyed indicated that PPI groups made either ‘some contributions’ or ‘significant contributions’ to the HOMESIDE study both nationally and internationally. The national groups were slightly more often rated as having made ‘significant contributions’ (57.1%) to the study, whereas the international group was more often rated as having made ‘some contributions’ (57.1%). The PPI members were asked to indicate whether they had contributed in the ways they were hoping, and most PPI members responded ‘Unsure’ (62.5%); however, one-third responded that they had (31.3%). The majority of academics felt that the PPI input brought the research closer to the lived experience of dementia (71.4%), and most of the PPI members also felt that the study was connected to their own experiences with dementia (75%).

**Table 2:** Contribution of PPI to the HOMESIDE study

	Proportion, n (%)	
	PPI Members	Academics
**Do you feel your national HOMESIDE PPI group was able to contribute to the study?**		
Yes, the PPI group made significant contributions	N/A	4 (57.1%)
Yes, the PPI group made some contributions	N/A	3 (42.9%)
No, the PPI group didn’t make many or any contributions	N/A	0 (0.0%)
Unsure	N/A	0 (0.0%)
**Do you feel the international HOMESIDE PPI group was able to contribute to the study?**		
Yes, the PPI group made significant contributions	N/A	2 (28.6%)
Yes, the PPI group made some contributions	N/A	4 (57.1%)
No, the PPI group didn’t make many or any contributions	N/A	0 (0.0%)
Unsure	N/A	1 (14.3%)
**Do you feel that the PPI group has further connected the research to the lived experience of dementia?**		
Yes	N/A	5 (71.4%)
No	N/A	0 (0.0%)
Unsure	N/A	2 (28.6%)
**Do you feel the research and being involved in the PPI group is connected to your lived experience of dementia?**		
Yes	12 (75.0%)	N/A
No	1 (6.3%)	N/A
Unsure or No answer	3 (18.8%)	N/A
**Have you been able to contribute to the HOMESIDE study in the ways you were hoping?**		
Yes	5 (31.3%)	N/A
No	1 (6.3%)	N/A
Unsure	10 (62.5%)	N/A

The respondents were asked to expand on the PPI groups’ contributions nationally and internationally in free-text comments. Three themes related to contributions to the research study were identified:

1. Supporting recruitment and publicity

Both academic and PPI members discussed the value that PPI added to the research team’s communication with the public during the trial, especially support of participant recruitment-related publicity. Respondents highlighted PPI input into advertising and promotional materials, such as brochures, videos, emails, webinars, and social media posts, improved the accessibility of the language and design used. They were also involved in media appearances (for an example, see Additional file 7), presentations, and workshops to support dissemination and awareness. Academics noted that communication with the public was more effective and had the potential to reach more diverse groups because of PPI members’ contributions:

*‘They ensured our recruitment materials were inclusive and ... supported us in using language that was accessible and relevant to potential participants’ (Academic 6)*

Responses also acknowledged how PPI members were well-positioned to reach potential participants through pre-existing contacts and awareness of organizations. Importantly, PPI input into recruitment materials and events seemed to lead to increased overall uptake in study participation.

2. Contributing to the intervention design and delivery

A limitation of the trial’s PPI was the formation of the groups after the interventions and related materials had been designed; it was strongly felt that this would have been a valuable way for PPI members to contribute to the early development and design of the study. However, this did not preclude ongoing advice from PPI members regarding effective delivery of the study:

*‘Within the limitations of having the PPI group join the study after the intervention had been developed, I still think the group contributed to subtle ways the intervention could be delivered to participants to ensure it was relevant and helpful. For example, how to frame the activities, how to approach participants, etc.’ (Academic 6)*

Additionally, many respondents described how the move from in-person to online delivery after trial commencement created an opportunity to advise on delivery:

*‘[support] the research team, most of all during the implementation of the online version of the project’. (PPI member 12)*

Encouragement from PPI members during this unexpected and difficult period was valued by the research team, who had been hesitant to move the study online. Thus, the study successfully transitioned to online delivery, drawing from the insights and suggestions provided by the PPI members.

3. Identifying cultural differences

PPI members reflected on the sharing and learning related to cultural differences as a key achievement of the international meetings:

*‘learning through comparison how dementia services work in other countries and how music/reading therapy is promoted’ (PPI member 4)*

Consideration of cultural norms and differences regarding dementia care and the use of music or reading across countries created possibilities to learn and solve problems together, as well as identify where challenges were due to cultural differences.

### Impact on individuals

Table 3 presents respondents’ perceptions of how PPI in the HOMESIDE trial impacted their understanding of dementia and how they approach dementia care. PPI members were more likely to report an increased understanding of dementia and dementia research (62.5%) than academics (42.9%), whereas academics were more likely to feel that PPI changed their relationships with the people with dementia with whom they work (42.9% of academics versus 18.6% of PPI members). PPI members were more likely to report that their involvement led to an increase in their creativity and approaches when caring for someone with dementia (50% of PPI members versus 14.3% of academics). Being involved in the study for the most part did not impact PPI members’ self-reported stress levels (81.6%); however, two PPI members did report an increase in stress, where one indicated this was due to the increased time investment as the chair for their national group.

**Table 3:** Impact of PPI involvement on PPI members and academics as individuals

	Proportion (n, %)	
	PPI Members	Academics
**Has your involvement in PPI changed or improved your understanding of dementia or dementia research?**		
Yes	10 (62.5%)	3 (42.9%)
No	6 (37.5%)	4 (57.1%)
Unsure	0 (0.0%)	0 (0.0%)
**Has your involvement in PPI changed your relationship with your carer, the person you care for, or the people with dementia that you work with?**		
Yes	3 (18.6%)	3 (42.9%)
No	9 (56.3%)	3 (42.9%)
Unsure	4 (25.0%)	1 (14.3%)
**Has your involvement in PPI expanded your creativity, thinking or approaches when caring for someone with dementia?**		
Yes	8 (50.0%)	1 (14.3%)
No	2 (12.5%)	2 (28.6%)
Unsure or No answer	6 (37.6%)	4 (57.2%)
**Has your involvement in PPI impacted your stress levels?**		
Significantly decreased my stress levels	0 (0.0%)	N/A
Somewhat decreased my stress levels	1 (6.3%)	N/A
Neither decreased or increased my stress levels	13 (81.6%)	N/A
Somewhat increased my stress levels	2 (12.5%)	N/A
Significantly increased my stress levels	0 (0.0%)	N/A

In the free-text comments, respondents also spoke about how being involved in PPI impacted themselves and their work more broadly. The following six themes were identified related to the impact on PPI members and academics as individuals:

1. Meeting others, sharing experiences, and receiving support

Some respondents reported that being part of the PPI group created opportunities for meeting others as well as building a wider network for people living with dementia and their caregivers. This created opportunities for sharing their own experiences of living with dementia or caregiving, working in the field, using music, or participating in the study, and this contributed to a mutual understanding between members:

*‘You share experiences and the nuances of caring with others who understand’ (PPI member 4)*

PPI members reported that they felt they could openly share their experiences, and this contributed to mutual understanding and a sense of support within meetings.

2. Learning about dementia and research

Both PPI members and academics discussed the impact of PPI on their learning. Some gained new perspectives on dementia and could see the realities of dementia in a new light:

*‘it allows me to think about working/living with dementia differently’ (PPI member 1)*

Others commented on how being involved contributed to their understanding of how dementia research is carried out and that it increased their interest in seeking answers through research. One PPI member highlighted that part of the learning experience was recognizing their own value in the research process:

*‘learned that non-academic researchers can give meaningful input’ (PPI member 3)*

Academics described how working with people with lived experiences expanded their own understanding of what matters to people affected by dementia and the value of dementia research in affecting change.

3. Changes in own use of music and reading

Several respondents reflected on how their involvement in PPI changed the ways in which they used music or reading when caring for someone with dementia. Some identified that their use of these activities increased, and their repertoire of activities expanded:

*‘increased...variety of songs and books I share with my loved one’ (PPI member 3)*

One PPI member also commented on integrating these changes into their professional life, as they learned new therapeutic techniques that they could use in their daily work with people with dementia.

4. New experiences

Being involved in the research process created novel and unexpected experiences for PPI members, and emphasized how overall being involved in research was a new experience for them:

*‘keeping that ability to learn and participate in “new” stuff is exciting and motivating’ (PPI member 1)*

One member described how their involvement with the study led them to speak on the radio to promote the study, which was an unexpected and exciting experience that was initiated through being a PPI member.

5. Grounding the academic researchers in lived experiences

Several academic respondents commented on how interactions with the PPI group grounded them in lived experiences of people affected by dementia. Enhancing academics’ understanding of dementia was described as a key outcome of the PPI, which was achieved through sharing stories and experiences. The importance of maintaining this focus throughout the trial was highlighted as an achievement:

*‘[It was] keeping the researchers “down to earth” and always reminding them who this project is made for’ (Academic 5)*

One academic described an increased awareness of the realities of dementia, including the wider impacts on families and daily lives, and what is important to people affected by dementia.

### Challenges

Respondents were asked to reflect on the challenges and barriers to participation or achievements, both nationally and internationally. Several challenges were raised that impacted the potential contributions of PPI members. Free-text responses identified the following five key challenges:

1. Lack of awareness of purpose, roles, or objectives

Both academics and PPI members reflected on having a lack of awareness of the purpose, role, or objectives of PPI, especially at the beginning but also throughout the study. Some academics felt unsure of how to set up or facilitate the PPI groups without clear guidance or instructions. One researcher described that this was a shared feeling between them and the PPI members:

*‘...I know many PPI-members had the feeling of not knowing why they had been involved or what was expected of them, and as a team member I was not as able to meet this confusion as well as I would have wanted to.’ (Academic 1)*

This lack of clarity was echoed by PPI members, who at times felt unsure of the goals and objectives of the group, which made it difficult to contribute or participate:

*‘I think it has been a bit frustrating not being able to participate that much. I did not actually know why I was there’ (PPI member 15)*

2. Lack of opportunity to contribute to the trial design

The survey highlighted a shared sentiment that PPI members should have been involved much earlier in the research design:

*‘A new PPI group was developed for the study, which was already designed and funded – this impacted how much they could contribute to the design’ (Academic 6)*

Although public consultations took place at the study’s funding application stage, the members of the formal PPI groups had mostly not been involved at that stage; this led PPI members to feel unsure about how they could contribute. As the study was already designed when they became involved, some PPI members expressed that there were few opportunities to support the research team in key decisions.

3. Meeting online versus face-to-face

Several respondents discussed challenges that arose when PPI meetings moved from face-to-face to online due to the COVID-19 pandemic, which included difficulties participating, lack of direct contact, and lost nuance. Members described challenges getting to know each other online, some having often met only once in-person prior to the pandemic:

*‘The online-only formula limited natural relationships and was a barrier to further involvement’ (PPI member 12)*

Despite such challenges, one member felt it was much easier for them to meet on Zoom than in-person and some acknowledged that, given the international nature of the study, meeting online was appropriate.

4. Scheduling around time commitments and time zones

Several factors were raised that contributed to scheduling issues for meetings, including working across time zones as well as family and work commitments for PPI members. Several PPI members were also professionals, so meetings scheduled during working hours made it difficult to participate. These scheduling issues could limit how much members could attend and contribute, particularly to international meetings:

*‘Owing to the range of time zones and PPI members being working members of society, it was quite rare that meetings could be attended across all countries. This seemed to stunt some progress in the international group’ (Academic 7)*

5. Language barriers

Language barriers were also highlighted by PPI members and academics as a challenge that could limit PPI members’ attendance and participation in international meetings, whether these were specifically for PPI purposes or when invited to join the research team’s full-team meetings:

*‘some participants were reluctant to join the international meetings as they needed to speak English’ (PPI member 7)*

There was a sense that language barriers could negatively impact on collaboration, as participants who did not speak English could be hesitant to attend or participate.

## Discussion and recommendations

Our findings identify the benefits and contributions of PPI within the HOMESIDE trial, while also highlighting the challenges that arose. To address these challenges, we developed recommendations for future PPI in dementia research based on the above findings, our survey respondents’ free-text responses to questions regarding the future of PPI, and the previous literature. The recommendations are meant to be flexible and non-prescriptive, recognizing the need for varied approaches to PPI to meet the needs of the research and to reach a diverse range of PPI members [4, 8, 15, 16]. Five recommendations were identified:

1. Involve PPI members as early as possible and throughout the research process

People affected by dementia should be involved throughout the research process, from the development of research ideas through to implementation and dissemination, as articulated by a PPI member:

*‘PPI should be involved in study design, recruitment and the development of participant materials right from the start and acknowledged in the results of the research.’ (PPI member 7)*

Specific to the HOMESIDE study, both PPI members and academics felt that the PPI could not contribute in the way they had hoped due to the formal PPI groups being established after funding had been secured and details of the study had been decided. This emphasizes the importance of embedding a co-design process when designing studies and interventions [29] and maintaining the consistency of PPI members throughout the research cycle of a project. Furthermore, respondents in our survey highlighted that they would like to see an increase in people affected by dementia leading research themselves.

To enable the inclusion of PPI throughout the research process, research groups may benefit from the establishment of sustainable PPI by seeking funding from PPI-specific funding bodies or calls. Since HOMESIDE, the music therapy and dementia research group at Anglia Ruskin University have formed a permanent Lived Experience Advisory Panel (LEAP) through institutional funding dedicated to increased public involvement in research. The LEAP is involved in developing priorities for research and earlier stages of research design. This approach may help to bridge the gap in public involvement between different stages of the research lifecycle and has also been implemented in other research centres in the UK, such as the Centre for Ageing and Dementia Research [30].

2. Create a space for constructive criticism and feedback

Our findings emphasize that for PPI to be effective, there is a need to develop trusting relationships with the research team early in the research process. This can create a safe space and therefore opportunities for PPI members to provide honest, constructive feedback to academic team members. A willingness to receive feedback is also essential:

*‘be open to constructive criticism and feedback from those with lived experiences – it will make your research better’ (Academic 6)*

We found that opportunities to meet in-person, rather than only online, may be important and can help to facilitate early relationship building across PPI members and academics.

A safe space and strong rapport can help facilitate two-way learning between PPI members and academics, which has previously been described as attaining ‘experiential knowledge’ [4]. This sense of ‘grounding the academic researchers in lived experiences’ was identified as one of the impacts of PPI within the HOMESIDE study, where academics’ ideas, values, and assumptions were challenged through this gained insight. A respondent emphasized the value of PPI members becoming involved in research:

*‘academics value your experience and need your point of view to link theoretical ideas with real life experiences’ (PPI member 7)*

This form of learning, through direct dialogue between academics and those affected by dementia, would be difficult to replicate through less direct approaches or if power dynamics influence open conversations [6].

3. Have clear tasks, roles, and expectations for PPI members

The findings highlight the importance of the research team managing expectations and facilitating respectful and effective PPI by identifying clear tasks, roles, and expectations for PPI within each stage of the study. Previous literature has emphasized the misunderstandings and tension that can be caused when there is a mismatch between PPI members’ and academics’ expectations [8]; this may have contributed to some of the feelings of frustration experienced by the PPI members in the HOMESIDE trial. Overall, it is important that PPI meetings are purposeful and that expectations are outlined to help facilitate transparency:

*‘Ensure there is clarity about what is expected from PPI members and that there is always a clear purpose for meetings (valuing members’ time)’ (Academic 6)*

From our survey, recommendations to support this included having a mission statement for PPI input from the outset, as well as providing written information before and after meetings to allow time for contemplation and preparation. Tasks should be identified by taking into consideration the distinct knowledge and skills of both PPI members and academics [17]. Training needs for PPI members should also be considered, which could cover topics such as the research cycle, funding applications, research design, developing research questions, or specific research methods; this could increase PPI members’ understanding of roles, goals, and expectations for the project.

4. Involve PPI members with a diverse range of experiences and backgrounds

Our survey respondents noted that they would like to see more diversity in PPI membership, including current carers, a wider age range or mix of age representation, young carers, and people with a diagnosis of dementia:

*‘greater involvement from people who have dementia’ (PPI member 3)*

A lack of diversity could unintentionally contribute to health inequalities, as identified in previous literature [6], where certain perspectives are emphasized while omitting perspectives from other groups or experiences. Careful consideration of how to approach and engage potential PPI members, including being flexible in using both formal and informal approaches [15] and managing language barriers, is necessary to involve a range of perspectives.

To diversify PPI membership, research groups should use a variety of approaches to recruit PPI members, including meeting with diverse established dementia groups or engaging with people with lived experience on social media. Potential language barriers should be considered early on and, particularly for international trials, specific plans to address language barriers should be implemented. Meetings can be planned and carried out based on the needs and preferences of the members, taking into consideration the length and time of meetings and, if necessary, the use of live translators. Members may also have varying preferences for giving feedback, therefore academics should allow for feedback in meetings, small group or individual discussions, or by e-mail.

5. Embed infrastructure and planning to support PPI

Similar to previous literature, these findings emphasize the funding and time required for meaningful PPI input [10]; appropriate practical and financial infrastructure has the potential to widen access to participation and improve the effectiveness of PPI. In our survey, one academic recommended that there should be a PPI lead or designated individual responsible for PPI embedded into research teams, who could be responsible for facilitating communication and addressing training needs for PPI members:

*‘paid PPI positions to improve links between “academics” and communities affected’ (Academic 7)*

Furthermore, access to funding to support PPI needs to be available throughout the research cycle, from the development of research questions, during funding applications, throughout study implementation, and for dissemination. This should include the offer of reimbursement for PPI members’ time, showing monetary value for their time, expertise, and knowledge. The National Institute for Health and Care Research (NIHR) payment guidelines [31] can support researchers to set rates and consider arising issues such as impact on individuals’ benefits and taxable income. When working cross-culturally, funding may also be needed to support translators, including live translators during meetings.

In multi-site and international studies, challenges in implementing PPI arise due to differing levels of experience across countries and research groups. Hence, it is crucial to develop a PPI strategy early in the research process, which can be led by experienced PPI lead. This strategy should include training for less experienced research groups.

### Limitations

Due to the nature of the current study, there are limitations that need to be addressed. The survey explored the experiences of PPI within a single study, potentially limiting its generalizability. Further, although the survey was co-produced with a PPI co-researcher, the analysis was carried out by academic researchers and PPI members may have interpreted qualitative findings differently.

The inability to collect specific demographic information presents a risk that the responses may not be representative of the whole group. Regarding PPI members, although we received responses from a balanced mix of individuals with personal and professional connections to dementia, we lack information on how many respondents had diagnosis of dementia and it is therefore possible we did not capture their views. The low response rate from academics may mean that the views captured are from those most involved in PPI, possibly meaning the findings are biased towards positive views of PPI.

The lack of demographic data also prevented us from identifying country differences across the international PPI groups. However, we hope that this limitation is partially mitigated by having co-authors from all countries, ensuring the recommendations are relevant across our diverse contexts.

## Conclusion

This paper outlines an example of PPI in practice across a large-scale international research study for people with dementia and their family caregivers. The impact of PPI within the study was wide-ranging, including contributions to practical study procedures and implementation, and benefits to PPI members and academics through opportunities for learning and connecting. The findings emphasize the importance and value of embedding PPI input across all research stages, specifically highlighting the impact on research delivery and supporting participant recruitment to achieve large sample sizes. However, the findings also indicate challenges in implementing PPI, especially across multiple international research sites over many years, and the recommendations were developed to address these challenges. The experiences of PPI within HOMESIDE, based on these findings, indicate areas for improving the implementation and effectiveness of PPI.

### Supplementary Information

**Supplementary Material 1.**

**Supplementary Material 2.**

**Supplementary Material 3.**

**Supplementary Material 4.**

**Supplementary Material 5.**

**Supplementary Material 6.**

**Supplementary Material 7.**

## Data Availability

The datasets used and analyzed for the current study are available from the corresponding author on reasonable request.

## Abbreviations

HOMESIDE: HOME-baSed family caregiver-delivered music and reading Interventions for people living with Dementia
JPND: EU Joint Programme – Neurodegenerative Disease Research
PPI: Participant and Public Involvement
RCT: Randomized Controlled Trial

## Footnotes

1 HOMESIDE was supported by the EU Joint Programme – Neurodegenerative Disease Research (JPND), which included joint funding from the National Health and Medical Research Council, Australia (APP1169867); The Research Council of Norway (project no. 298995); Federal Ministry of Education and Research, Germany (01ED1901); The National Centre for Research and Development, Poland (JPND/04/2019); and Alzheimer’s Society, UK (grant no. 462).
