# Supplementary material for: Experiences of participant and public involvement in an international randomized controlled trial for people living with dementia and their informal caregivers
Source: Res Involv Engagem. 2024 May 2;10:43. doi: 10.1186/s40900-024-00574-2 (PMC11064380; doi:10.1186/s40900-024-00574-2)
Supplement: Supplementary file 1 — Supplementary Material 1. [file 40900_2024_574_MOESM1_ESM.pdf]

a.r.u. | Cambridge Institute for Music Therapy Research

Alzheimer's Society United Against Dementia

Invitation to participate in a research study

Homeside

Music and reading for people with dementia and their caregivers

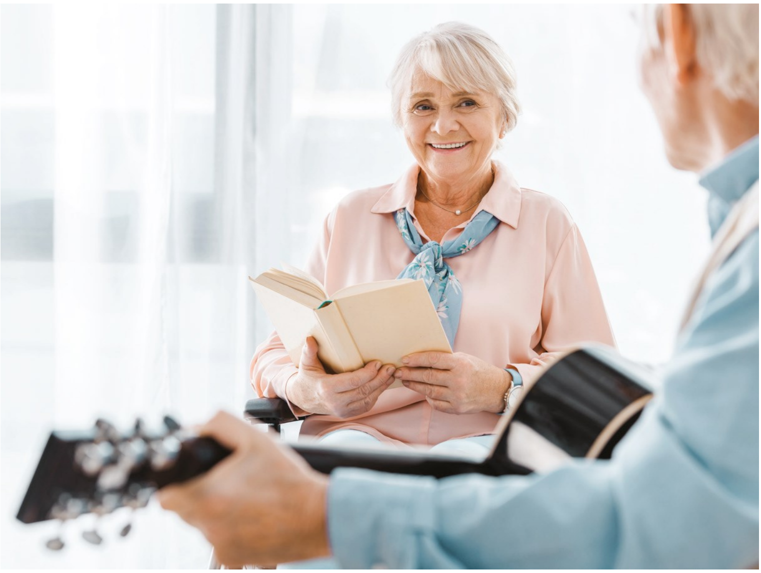

Homeside
Partnership in dementia care

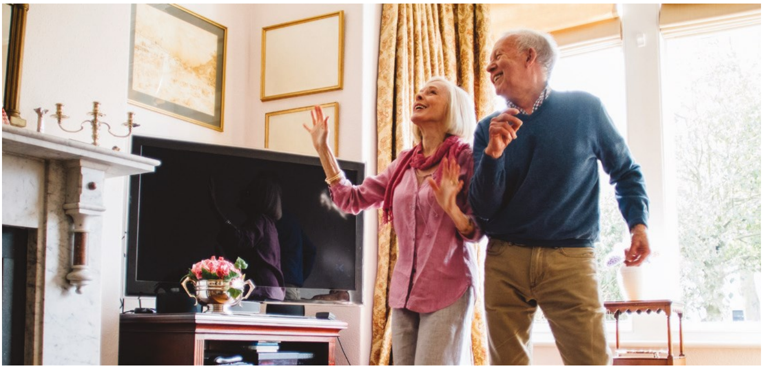

What is the focus of this study?

Homeside will investigate the effects of music and reading activities for people living with dementia and their family caregivers.

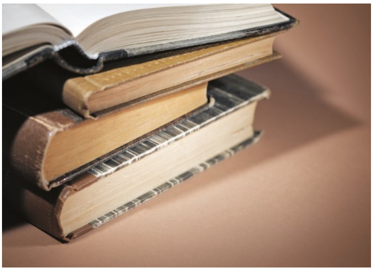

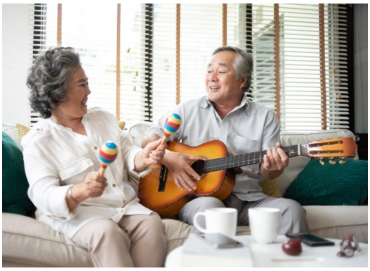

Who is the study for?

This study is for people living with dementia and their family (or informal) caregivers. You can participate in this study if:

- You or your relative have a diagnosis of dementia or show signs of dementia
- You are or you have a family caregiver
- You live together at home

No prior music or reading skills are required to take part!

What can participants expect?

During the Homeside research project:

- Everything will take place in your own home
- You will participate in the study for 6 months
- Caregivers will receive training from experienced professionals to use music or reading activities at home with the person they care for
- You both will be asked to fill out questionnaires at 3 points: at the beginning, after 3 months and after 6 months.
- Contact with researchers will take place using a secure video telehealth platform

The music and reading programmes will:

- Be tailored to your individual preferences and needs
- Offer opportunities to try and improve (new) skills and share meaningful moments
- Offer opportunities to sing together, to listen to music and stories, to dance, to

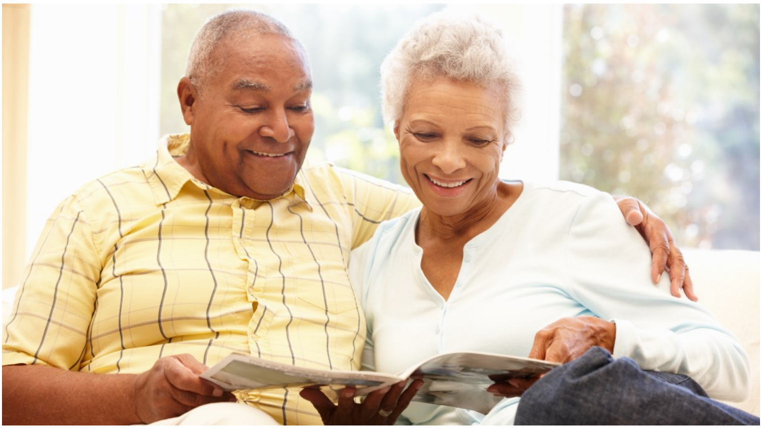

Why are we conducting this study?

Previous research indicates that such activities increase wellbeing, strengthen relationships, and improves care. In our study, Homeside, this effect is investigated comprehensively for the first time.

The study is an international collaboration, supported in the UK by Alzheimer's Society.

How is the study conducted?

When you participate in this study, you will be randomly allocated to either a:

- Music programme
- Reading programme
- Control group (no programme)

The programmes are designed to be delivered by family caregivers, who will receive training in their own home using an online telehealth video platform.

If you are allocated to the control group, you can choose to access the music or reading programme after your study participation free of charge.

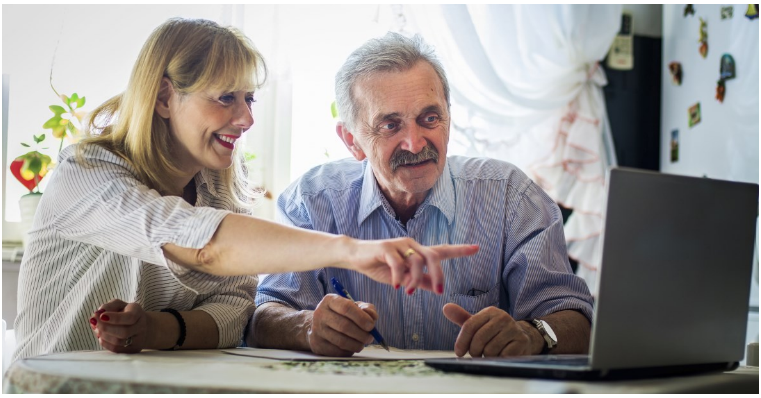

Share memories.

Enjoy common interests.

Use and build skills.

Manage symptoms of dementia.

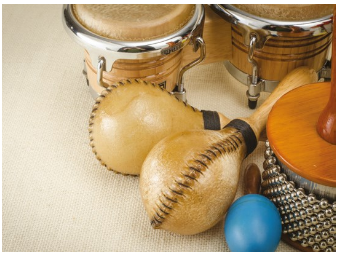

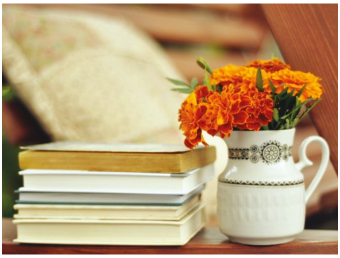

Are you interested in participating in this study or would you like to receive more information? Please contact us to register or to ask any questions you may have.

Contact:

Dr Ming-Hung Hsu or **Dr Jonathan Pool**

Tel: 01223 695401

Email: homeside@aru.ac.uk

Website: www.aru.ac.uk/homeside

The Cambridge Institute for Music Therapy Research (CIMTR)

Anglia Ruskin University

East Road, CB1 2PT

a.r.u. | Cambridge Institute for Music Therapy Research

Alzheimer's Society United Against Dementia

JPND research EU Joint Programme – Neurodegenerative Disease Research

IRAS I.D. 260067 ● Photos: Colourbox.com / Shutterstock.com
