# Supplementary material for: Experiences of participant and public involvement in an international randomized controlled trial for people living with dementia and their informal caregivers
Source: Res Involv Engagem. 2024 May 2;10:43. doi: 10.1186/s40900-024-00574-2 (PMC11064380; doi:10.1186/s40900-024-00574-2)
Supplement: Supplementary file 5 — Supplementary Material 5. [file 40900_2024_574_MOESM5_ESM.pdf]

Survey questions for PPI members (downloaded from Jisc Online Surveys)

Page 3: Background

What is your relationship to dementia? (choose all that apply)

- ☐ Personal
- ☐ Professional
- ☐ Other

How did you hear about the HOMESIDE PPI? (Choose all that apply)

- ☐ HOMESIDE Participant
- ☐ Involved in previous study with the research team
- ☐ Word-of-mouth
- ☐ Via another organisation (for example, Alzheimer's Society)
- ☐ Other

If other, please comment.

Why did you join the HOMESIDE PPI group? (Choose all that apply)

- ☐ Benefit for others with dementia
- ☐ Benefit for self/family/friend
- ☐ Personal interest in music
- ☐ Personal interest in reading
- ☐ Other

Please expand if you would like

Page 4: Experience and Contributions

On a scale of 1-5 (where 1=lowest and 5=highest), how involved did you feel in the HOMESIDE Study?

- 1 (Not at all involved)
- 2
- 3
- 4
- 5 (highly involved)
- Unsure

Please expand if you would like

How frequently were you able to attend the PPI meetings?

- None of the meetings
- A few meetings
- Half the meetings
- Most of the meetings
- All the meetings

What were your expectations of the PPI group? Were these expectations met?

		Yes	No	Unsure	Comments
Expectation 1					
Expectation 2					
Expectation 3					
Expectation 4					

Expectation 5		◌	◌	◌	

Please expand if you would like

Do you feel that the PPI group collaborated well with the academics involved in the Homeside Study?

- ◌ Yes, nationally (own country's Homeside team)
- ◌ Yes, internationally (entire international Homeside team)
- ◌ Yes, nationally and internationally
- ◌ No
- ◌ Unsure

Please expand if you would like

Have you been able to contribute to the Homeside Study in the ways you were hoping?

- ◌ Yes
- ◌ No
- ◌ Unsure

Please expand if you would like

	What were your main contributions to the PPI?
Nationally	

Internationally	

	What were the main achievements of the PPI?
Nationally	
Internationally	

Did COVID impact your involvement in or experience of the PPI?

- Yes
- No
- Unsure

Please expand if you would like

Do you feel you were adequately compensated for your time and input as a PPI member in the Homeside Study?

- Yes
- No
- Unsure

Please expand if you would like. (If no, what do you feel would be adequate compensation?)

Page 5: Effects of the PPI

Has your involvement in PPI changed your relationship with your carer, the person you care for, or the people with dementia that you work with?

- Yes
- No
- Unsure

Please expand if you would like (If Yes, what changed about your relationship?)

Has your involvement in PPI changed or improved your understanding of dementia or dementia research?

- Yes
- No
- Unsure

Please expand if you would like. (If Yes, what changed or what did you learn about dementia or dementia research?)

Has your involvement in PPI expanded your creativity, thinking or approaches when caring for someone with dementia? (For example, increased your use of music or the arts or given you new ideas)

Do you feel the research and being involved in the PPI group is connected to your lived experience of dementia?

Has your involvement in PPI impacted your stress levels?

- Significantly decreased my stress levels
- Somewhat decreased my stress levels
- Neither increased nor decreased my stress levels (no impact on my stress)
- Somewhat increased my stress levels
- Significantly increased my stress levels

Please expand if you would like

Page 6: Future of PPI

What do you feel the challenges or barriers were for the Homeside PPI group (for either participation or achievements)? What didn't work well?

	Challenges or barriers for the Homeside PPI group
Nationally	
Internationally	

Do you feel the frequency of meetings was appropriate?

- Yes
- No
- Unsure

Please expand if you would like

What is your preference for meeting format?

- In-person
- Online
- Hybrid

Please expand if you would like

What would you recommend is continued or done differently in future PPI groups?

What do you think of the term 'Patient and Public Involvement'? Is there a title or term you think would be better?

What change would you like to see over a generation in relation to PPI in dementia research?

If someone was considering participating in a PPI group, what would your recommendations to them be?

Is there anything else you'd like to say about your experience of being involved in the PPI group?
