# Supplementary material for: Experiences of participant and public involvement in an international randomized controlled trial for people living with dementia and their informal caregivers
Source: Res Involv Engagem. 2024 May 2;10:43. doi: 10.1186/s40900-024-00574-2 (PMC11064380; doi:10.1186/s40900-024-00574-2)
Supplement: Supplementary file 6 — Supplementary Material 6. [file 40900_2024_574_MOESM6_ESM.pdf]

Survey questions for academics (downloaded from Jisc Online Surveys)

Page 3: Experience and Contributions

On a scale of 1-5 (where 1=lowest and 5=highest), how involved did you feel in the HOMESIDE PPI?

- 1 (Not at all involved)
- 2
- 3
- 4
- 5 (highly involved)
- Unsure

Please expand if you would like

How frequently were you able to attend the PPI meetings?

- None of the meetings
- A few meetings
- Half the meetings
- Most of the meetings
- All the meetings

What were your expectations of the PPI group? Were these expectations met?

		Yes	No	Unsure	Comments
Expectation 1					
Expectation 2					
Expectation 3					
Expectation 4					

Expectation 5		○	○	○	

Please expand if you would like

Do you feel that the academics collaborated well with the PPI Members involved in the Homeside Study?

- ○ Yes, nationally (own country's Homeside team)
- ○ Yes, internationally (entire international Homeside team)
- ○ Yes, nationally and internationally
- ○ No
- ○ Unsure

Please expand if you would like

Do you feel your **national** Homeside PPI group was able to contribute to the study?

- ○ Yes, the PPI group made significant contributions to the study
- ○ Yes, the PPI group made some contributions to the study
- ○ No, the PPI group didn't make many contributions to the study
- ○ No, the PPI group didn't make any contributions to the study
- ○ Unsure

Please expand if you would like

Do you feel your **international** Homeside PPI group was able to contribute to the study?

- Yes, the PPI group made significant contributions to the study
- Yes, the PPI group made some contributions to the study
- No, the PPI group didn't make many contributions to the study
- No, the PPI group didn't make any contributions to the study
- Unsure

Please expand if you would like

	What were your main contributions to the PPI?
Nationally	
Internationally	

	What were the main achievements of the PPI?
Nationally	
Internationally	

Did COVID impact the PPI group in your country?

- Yes
- No
- Unsure

Please expand if you would like

Page 4: Effects of the PPI

Has your involvement in PPI changed your relationship with the people with dementia that you work with?

- Yes
- No
- Unsure

Please expand if you would like (If Yes, what changed about your relationship?)

Has your involvement in PPI changed or improved your understanding of dementia or dementia research?

- Yes
- No
- Unsure

Please expand if you would like. (If Yes, what changed or what did you learn about dementia or dementia research?)

Has your involvement in PPI expanded your creativity, thinking or approaches when caring for someone with dementia? (For example, increased your use of music or the arts or given you new ideas)

Do you feel that the PPI group has further connected the research to the lived experience of dementia?

- Yes
- No
- Unsure

Page 5: Future of PPI

What do you feel the challenges or barriers were for the Homeside PPI group (for either participation or achievements)? What didn't work well?

	Challenges or barriers for the Homeside PPI group
Nationally	
Internationally	

Do you feel the frequency of meetings was appropriate?

- Yes
- No
- Unsure

Please expand if you would like

What is your preference for meeting format?

- In-person
- Online
- Hybrid

Please expand if you would like

What would you recommend is continued or done differently in future PPI groups?

What do you think of the term ‘Patient and Public Involvement’? Is there a title or term you think would be better?

What change would you like to see over a generation in relation to PPI in dementia research?

If someone was considering setting up a PPI group, what would your recommendations to them be?

Is there anything else you'd like to say about your experience of being involved in the PPI group?
